# How do pharmacists navigate clinical uncertainty when reviewing polypharmacy? A critical literature review

**DOI:** 10.1186/s12875-025-03122-3

**Published:** 2025-11-27

**Authors:** Tomazo J. Kallis, Karen Mattick, Jenny Scott, Rupert A. Payne

**Affiliations:** 1 Department of Health & Community Sciences, Faculty of Health and Life Sciences, University of Exeter, Exeter, UK; 2 Centre for Academic Primary Care, Population Health Sciences, Bristol Medical School, University of Bristol, Bristol, UK

**Keywords:** Clinical pharmacists, Deprescribing, Polypharmacy, Primary health care, Uncertainty

## Abstract

**Background:**

Clinical pharmacists are the principal profession reviewing polypharmacy in English general practice. Pharmacists reviewing polypharmacy can encounter clinical uncertainty, thus affecting decision-making and the utility of medication reviews. Understanding factors and interventions that mitigate clinical uncertainty could improve polypharmacy medication reviews. This review’s objective was to explore how primary care clinical pharmacist decision-making can be improved when reviewing polypharmacy in the context of clinical uncertainty.

**Methods:**

A critical literature review was undertaken in key databases. Included articles explored polypharmacy, clinical uncertainty and medication review by primary care clinical pharmacists. Exclusion criteria included community pharmacy and monotherapy management. Quality assurance was conducted using Lincoln and Guba’s evaluative criteria. Contents of included papers were thematically analysed and conceptual models produced.

**Results:**

647 titles/abstracts were screened and 11 full-text articles included, encompassing focus group, interview, ethnography and intervention-development studies. Pharmacists expressed feelings of self-competence occurring alongside apprehension when reviewing polypharmacy. Relationships with patients, including shared decision-making, continuity of care and engagement can support medication reviews. Decision-making is impacted by environmental factors, with poor working relationships within organisations, working across several sites and time pressures hindering deprescribing. The absence of clinical and deprescribing guidelines for multimorbid patients contributes to clinical uncertainty. Multidisciplinary working mitigates clinical uncertainty and training interventions can support pharmacists to proactively deprescribe.

**Conclusion:**

Pharmacist, patient and environmental factors can influence pharmacists’ decision-making when experiencing clinical uncertainty during polypharmacy review. Clinical education, peer support and multidisciplinary working have roles in reducing clinical uncertainty and therefore optimising pharmacists’ reviews of polypharmacy.

**Supplementary Information:**

The online version contains supplementary material available at 10.1186/s12875-025-03122-3.

## Background

Polypharmacy and multimorbidity remain significant challenges for national health systems, with inappropriate multiple drug prescribing being associated with avoidable hospital admissions and increased falls risk [1, 2]. Polypharmacy is strongly correlated with non-adherence, decreased functional status and cognitive impairment [3]. Whilst polypharmacy may not be inherently harmful, the prescribing of multiple medications increases the likelihood of inappropriate or problematic polypharmacy, where the intended benefit of prescribed medication is not fully realised [4]. Although some evidence suggests medication review may reduce medication-related hospitalisation, evidence for interventions targeting polypharmacy is generally weak [5, 6]. The complexity of reviewing polypharmacy can compound clinical uncertainty. This uncertainty is a barrier to medicines optimisation, increasing the likelihood of clinicians opting to maintain the ‘status quo.’ [7].

A key element of national policy in England to address inappropriate polypharmacy has been the inception of the primary care network (PCN) clinical pharmacist role in 2019 to deliver Structured Medication Reviews (SMRs) in the general practice setting [8]. A priority group of patients eligible for an SMR are patients with ‘complex polypharmacy.’ [9] To support pharmacists in these new clinical roles, a nationally mandated postgraduate training pathway for all clinical pharmacists employed through PCNs was developed [10]. 

GPs and pharmacists report clinical uncertainty when reviewing complex, multimorbid patients [11]. Clinical uncertainty is poorly defined, but can be viewed as a function of ambiguity, probability and complexity, where the clinician has a “subjective perception of an inability to provide an accurate explanation of the patient’s health problem.” [12, 13] This uncertainty can present at any point in a clinical pathway. Individual clinician characteristics (such as experience, education, beliefs), as well as socio-cultural influences, can affect the perception and tolerance of clinical uncertainty by clinicians [14, 15]. Although the value of clinical training to actively explore ‘operating in a corridor of uncertainty’ to make patient-centred decisions is well known, educational interventions targeted at dealing with clinical uncertainty are infrequently delivered in undergraduate pharmacist training [16–18]. As a consequence, pharmacists can exhibit black-and-white thinking, treating clinical guidelines as ‘rules,’ and have cited needs for further support and training for clinical decision-making after qualifying [19–21]. 

This literature review aimed to explore how primary care clinical pharmacist decision-making can be improved when reviewing polypharmacy in the context of clinical uncertainty. The objectives to achieve this aim were to firstly understand how primary care clinical pharmacists make decisions in situations of clinical uncertainty when reviewing polypharmacy, and secondly to identify any interventions to support pharmacists experiencing clinical uncertainty when reviewing patients with polypharmacy.

## Methods

### Study design

A critical review of the literature was undertaken systematically in accordance with methods outlined by Booth et al. [22] Critical literature reviews seek to determine the most significant contributions in the field of interest, evaluating evidence by contribution as opposed to quality, and typically result in new conceptual models or hypotheses [22]. CINAHL, Embase, PsycInfo and MEDLINE databases were systematically searched using terms identified using a PICo framework (Table 1) [23]. 

**Table 1:** PICo framework detailing the population, phenomenon and context upon which database searches were based

PICo Domain	
Population	Clinical Pharmacists
Phenomenon of Interest	Polypharmacy, clinical uncertainty, medication review, deprescribing
Context	General Practice setting in primary care

### Registration

This literature review is part of the PPhoCUs (Polypharmacy, Pharmacists and Clinical Uncertainty) research programme, which is registered with ISRCTN [24]. A literature review protocol was developed and adhered to, which is publicly available; otherwise the review was not formally registered [25]. 

### Searching

Searches were built in OVID for PsycInfo, MEDLINE and Embase databases, and replicated in EBSCO for the CINAHL database (full search strategy in Supplementary Information). These searches were undertaken in December 2023, encompassing articles published in these databases from the previous 20 years (2003–2023). After de-duplication of returned results using the Zotero software package, articles (*n* = 647) were screened (TK) for inclusion via review of title and abstract. There were no restrictions on article type included for review, including editorials, evidence syntheses, and studies with both quantitative and/or qualitative approaches.

### Selecting studies and data extraction

10% of the returned articles were dual screened by an experienced senior researcher (RP) and a peer consensus meeting was held to agree any borderline papers. Table 2 describes the inclusion and exclusion criteria used for screening.

**Table 2:** Inclusion and exclusion criteria used for article screening in search strategy

Inclusion Criteria
Articles written in English
Primary care GP practice or PCN-based Pharmacists
Polypharmacy
Clinical Uncertainty
Primary Care Settings
Decision-Making
**Exclusion Criteria**
Exclusively other healthcare professional groups
Monotherapy or single morbidity management
Articles over 20 years old
Community pharmacists

Following initial title and abstract screening, full text review (TK) was undertaken to appraise articles for suitability for inclusion. Pertinent citations in included texts, were chased back and reviewed for inclusion also. A small number of articles at the full text review stage which were highlighted as having ambiguity for inclusion were discussed in a peer consensus meeting with the whole research team. Relevant conference and poster presentations were included in this review and where these met inclusion/exclusion criteria, author profiles were manually searched to identify any associated published articles.

Data were extracted from included papers (TK) using a modified tool based on the Joanna Briggs Institute mixed methods data extraction form, including author, year published, country, sample characteristics (population, size and setting), methodology, key findings and author conclusions [26]. The lead researcher (TK) also maintained a reflexive log of initial impressions and commentary on findings as data were extracted. Articles were reviewed for credibility, dependability, confirmability and generalisability/transferability, for quality assurance against the claims the articles made (see Supplementary Information) [27, 28]. Extracted data were collated using Excel and displayed in Table 3.

**Table 3:** A summary of characteristics and key findings of the articles included in this literature review

Author, Year, Country	Research Aim(s)	Study Design	Sample Characteristics	Key Findings
Birt et al. (2022) UK [29]	To explore beliefs and practices of deprescribing in care homes	Semi-structured interviews	16 pharmacists, 6 General practitioners and 7 care home managers	• Multiple actors are involved in the deprescribing process. • Pharmacists and GPs reflected that there was the potential for unintended consequences when deprescribing. • More complex, proactive deprescribing took place when care home staff knew a patient’s behaviours and needs. • Clinicians felt the balance of risks favoured stopping medication without robust clinical evidence in patients with frailty. • Pharmacists felt they were competent to deprescribe depending on their speciality. • Pharmacists were reluctant to deprescribe medication started in secondary care without contacting the prescriber. • Pharmacists expressed a desire for more peer support and formal guidance around deprescribing. • Variation between deprescribing activity in pharmacists was associated with whether the pharmacist had previously worked in their assigned GP practice.
Duncan et al. (2019) UK [30]	To explore GP and pharmacist perspectives on how medication reviews were conducted in general practice in the UK	Semi-structured interviews	13 General Practitioners and 10 pharmacists	• Participants perceived that pharmacists were more thorough when conducting medication reviews and that GPs were more time efficient. • Participants felt that frail patients did not need to be treated as aggressively for long term conditions. Barriers to stopping medicines included lack of clear evidence/guidelines, fear of causing problems and not wanting to stop medicines started in secondary care. • Multimorbidity and polypharmacy reviews were characterised as complex and prioritised for dedicated appointment slots. Pharmacists and some GPs highlighted that ‘proper’ medication reviews could not be done without patient involvement. • Participants felt patients should be involved in decisions about stopping medication. • Patients were characterised as often accepting harms of medicines if they improved their quality of life or wanting to continue medicines despite there being little evidence of benefit.
Ramsdale et al. (2023) USA [31]	To describe emotional barriers and facilitators to deprescribing in older adults with cancer and polypharmacy	Focus groups	6 Oncologists, 7 oncology nurses, 7 primary care physicians, 7 pharmacists and 9 patients	• Clinicians and patients universally agreed that polypharmacy was a problem needing intervention. • Pharmacists were apprehensive about reactions and moral judgments from colleagues when deprescribing. • Pharmacists also felt preoccupied about the reactions and moral judgments from patients, such as patients feeling abandoned or angry. • All groups viewed pharmacists as trustworthy, objective experts with the required skills to help facilitate deprescribing. • Pharmacists demonstrated an understanding of the system complexity and patient experience within that system in a way that the other healthcare professionals did not express. • Pharmacists reflected that their training equipped them well to make emotionally neutral recommendations to other healthcare professionals to help support deprescribing.
Ie et al. (2023) Japan [32]	To qualitatively explore the experiences and perspectives of general practitioners and pharmacists on deprescribing in multimorbid older adults, with the goal of developing effective patient-centred deprescribing strategies	Focus groups	16 Pharmacists and 19 General Practitioners	• Levels of comfort between participants to deprescribe varied. • Facilitators to deprescribing were given as multidisciplinary team (MDT) collaboration, partnership with pharmacies and continuity of care, whereas barriers included lack of incentives to deprescribe and loss of information between care settings. • Participants had positive attitudes towards deprescribing and expressed ‘strategic compassion,’ preferring to build relationships with patients over a period of time until they were ready to stop a medication. • Experience, education and environment shaped attitudes towards deprescribing. • There are subjective norms related to polypharmacy (peer pressure to deprescribe). • Drug factors associated with deprescribing included low adherence, unclear indications, ADR concerns, symptomatic drugs without clear benefits, and drug contraindications. • Prescriber factors encompassed clinical inertia (resistance to changing status quo), lack of openness to opinions from other clinicians, lack of partnerships or opportunities to collaborate. • Understanding of disease/treatment, good relationships with clinicians, difficulty in taking medicines, change in health status, adverse events, expectations and actively participating in reviews could all affect a patient’s willingness to discuss their medication.
Threapleton et al. (2020) England [33]	To develop a structured framework for specialist review of primary care patients with complex polypharmacy	Intervention development	Clinical pharmacologists, primary care clinicians and pharmacists	• Recommendations to stop or reduce doses of medication were more common for drugs used for symptom control or in prescribing cascades, as opposed to prophylaxis or disease control. • Renal function, U&Es, lipid profile and blood pressure were key tests required to inform decision making in the majority of polypharmacy work ups. • Using the Stopping By Indication Tool (SBIT) could potentially support deprescribing in primary care in the absence of specific guidelines.
Radcliffe et al. (2023) UK [34]	To develop a programme theory to inform recommendations for successful implementation of multidisciplinary deprescribing for older people within the context of primary care	Realist review, including scoping literature review, semi-structured interviews and focus groups	8 Practice pharmacists, 7 General Practitioners, 5 advanced nurse practitioners, 2 medical students, 2 frailty practitioners, 1 physiotherapist and 1 dietician	• Recommendation that pharmacists lead interventions, with GPs retaining final decision making. • When pharmacists lead medication reviews, there is more likely to be a reduction in inappropriate prescribing if well-integrated into an MDT. • When GPs are involved with meds review led by pharmacists, patients are more likely to accept any proposed changes. • There is a lack of clear clinical guidelines for deprescribing. • Education on deprescribing should run ‘from entry-to-practice’ and continue as part of ongoing professional development. • Full access to records and co-location of pharmacists in practices allowed clinicians to make better-informed decisions when deprescribing. Face to face medication reviews facilitate shared decision making. Patients with a high treatment burden are less likely to engage with an additional medication review appointment and it is recommended that meds reviews are aligned with other contemporaneous review activity. • Patients are more likely to accept recommendations from a trusted clinician, but are less likely to engage if they are anxious about recommendations.
Kirwan et al. (2022) ROI and NI [35]	To assess the feasibility of a definitive trial of the MyComrade intervention across two healthcare systems	Pilot cluster randomised controlled trial with semi-structured interviews	15 GP practices with both general practitioners and practice-based pharmacists.	• Both GPs and practice-based pharmacists commended the collaborative review approach used, citing that it ‘reduced uncertainty, critically appraised and corroborated treatments plans, increased both practitioner confidence and patient safety.’ • Patients welcomed having peer review of their medication reviews, believing it would improve the accuracy and appropriateness of prescribed medicines.
Madden et al. (2022) England [36]	To identify factors affecting the early implementation of the Structured Medication Review service	Semi-structured interviews	20 practice-based pharmacists	• Experienced pharmacists said SMRs were more timely than other types of review as they were more clinically complex. • Undergraduate pharmacy training encourages pharmacists to interpret guidelines as rules, meaning they were ‘ill equipped for the ambiguities in primary care practice that underpin shared decision making.’ • Pharmacists lack the patient-facing and ‘hands on’ training that doctors receive. • There was frustration around lack of support or perceived value of SMRs from GPs. • Newly appointed pharmacists relied more on consultation templates, whereas experienced pharmacists regarded these as tools to capture data rather than consultation structure tools.
Swinglehurst et al. (2022) England [37]	To illuminate how medication reviews are conducted in practice in the context of polypharmacy and elicit professional concerns about polypharmacy practices	Video Reflexive Ethnography	24 General practitioners, 5 practice nurses, 3 pharmacists and 2 practice managers	• Appointments for medication review often exceeded 10 min and were characterised as both mundane and complex. • Although recorded as discrete events, polypharmacy reviews stretched over several appointments with no clearly defined beginning or end. Appointments often overran or did not have sufficient time for clinicians to cover off all the associated problems. • Participants felt that polypharmacy should be tackled with small, incremental, carefully supported challenges. • Decisions about reducing or stopping medication were complex and potentially involved upsetting the status quo or ‘destabilising’ someone. The lack of evidence to guide action in polypharmacy reviews is highlighted, combined with concerns about responsibility and accountability in the face of inescapable uncertainty.
McCahon et al. (2022) England [38]	To explore patient experiences of medication review including the processes and activities that led up to and shaped the review	Semi-structured interviews	21 patients	• Patients felt uninformed as to the purpose of a medication review and developed their own rationales. • Patients reported feeling motivated attend when the appointment was timely, coinciding with medicines-related concerns or queries. • Clinicians were sometimes characterised as unprepared, apathetic and conducting a box-ticking exercise. • Patients felt that their experience would have been better if they understood the purpose of the review, with additional information sent beforehand. • There was a preference for a reviewer they knew and continuity of care, as well as for face-to-face encounters as opposed to telephone review.
Anderson et al. (2017) Australia [39]	To explore the views of GPs and clinical pharmacists about inappropriate polypharmacy and the reasoning they apply to deprescribing in primary care, and to identify factors that support or inhibit this cognitive process.	Focus groups	32 General Practitioners and 15 consultant pharmacists	• Clinicians cited difficulty in weighing up harms and benefits, even between similar patients - deprescribing activity could be affected by subtle differences such as care goals, likely future trajectory and patient function, all of which can change over time between and within individuals. • Inadequate research into polypharmacy in older adults also highlighted to support decision making. • Uncertainty could lead to inaction in when there is a paucity of evidence to support deprescribing. Clinicians engaged in gradual, incremental approach to deprescribing, prioritising ‘low hanging fruit.’ • GPs felt they had a lack of time, whereas pharmacists felt they had a lack of detailed knowledge about patients. • Clinician-patient relationships, as well was GP-pharmacist-specialist relationships were all cited as important. Concerns around deprescribing included unpredictability of outcome trajectory and fear of contributing to a worse outcome. • Deprescribing was seen as a risk to be reconciled when setting out to avoid future ADRs. • Falls, nonadherence, adverse events, direct requests from patients/carers to stop medicines all factored in pushing clinicians towards making deprescribing decisions, as opposed to maintaining the status quo.

### Synthesis

An integrated, qualitative approach to analysis was taken to allow for narrative synthesis of the reviewed literature [22]. Whilst the a priori categories of polypharmacy, clinical uncertainty and pharmacist-let interventions were anticipated to form the basis of the textual narrative, themes within these categories were identified during the synthesis of the literature. The ‘One Sheet of Paper’ method was used to inform a conceptual model to bring together themes identified in the analysed articles [40]. 

## Results

**Fig. 1:**
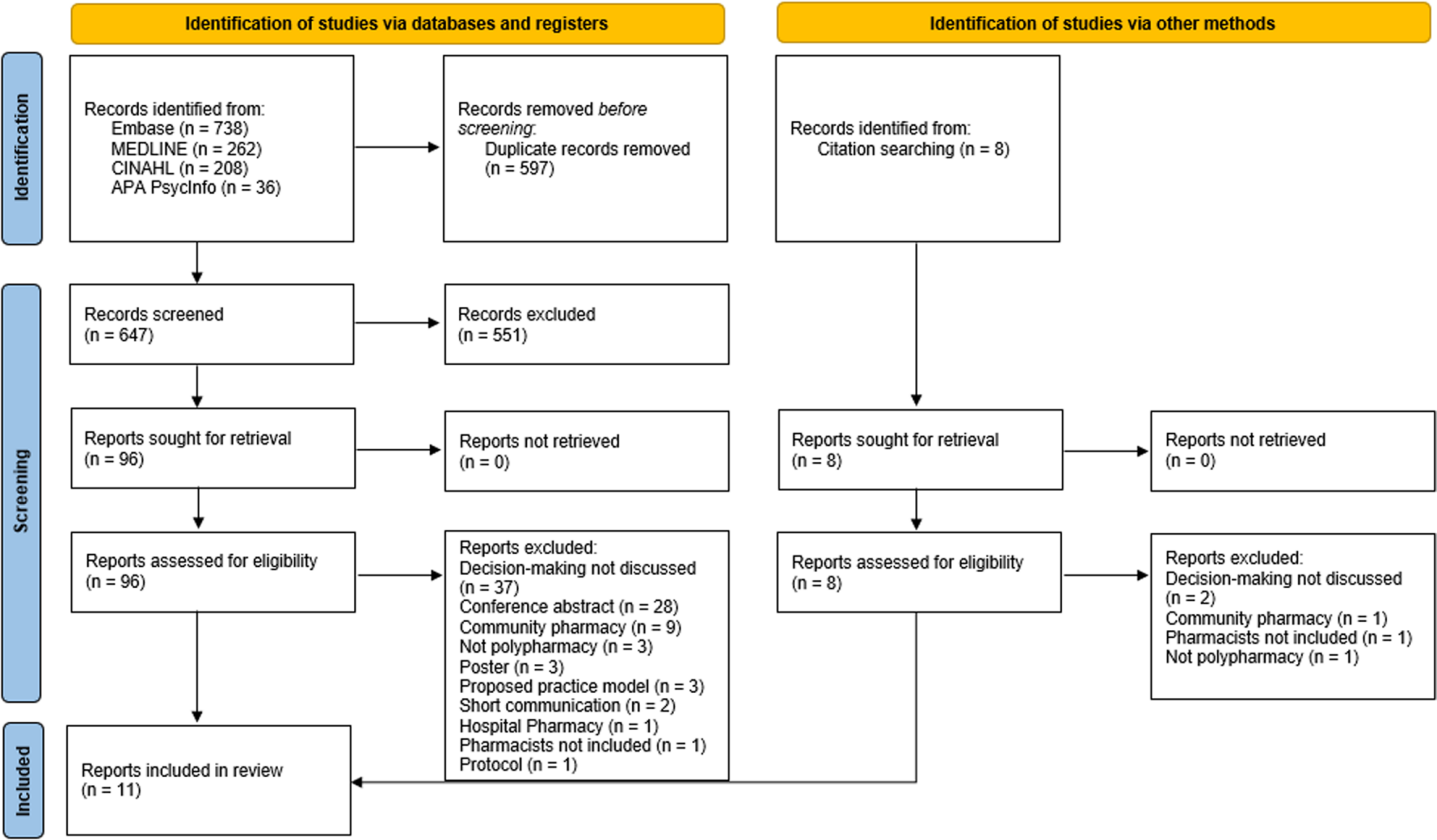
PRISMA diagram detailing identification and review of searched databases to yield included articles in this review

Electronic searches of the included databases returned 1,244 articles, with an additional 8 articles identified from citation searching. Eleven articles were selected for inclusion. Figure 1 displays the full screening process [41]. Of the eleven included articles, four were structured interview studies [29, 30, 36, 38], three used focus groups [31, 32, 39], one was a pilot cluster randomised controlled trial [35], one was an intervention development [33], one used realist review methodology [34] and one used video reflexive ethnography [37]. Eight of these studies were based in the United Kingdom (one of these also included sites in the Republic of Ireland) [29, 30, 33–38], with the remaining studies based in Australia [39], Japan [32] and the United States of America [31]. Table 1 in Supplementary Information gives a summary of study characteristics and key findings.

**Fig. 2:**
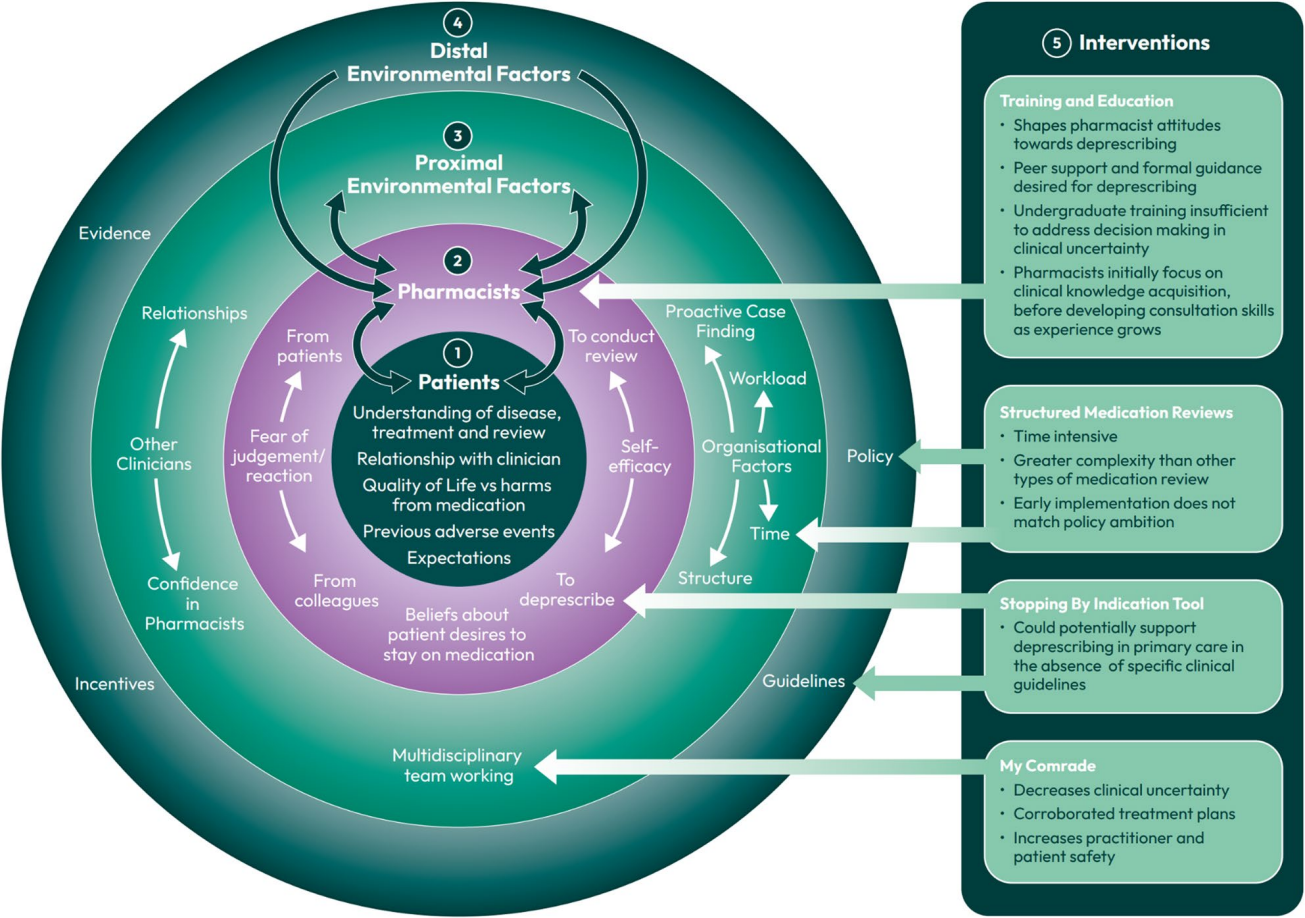
Factors affecting primary care clinical pharmacist decision making when reviewing polypharmacy in the context of clinical uncertainty. Patients (1) have their own psychological views of the medication review process and propensities to engage with the reviewing pharmacist. Pharmacists (2) will also have their own psychological and behavioural characteristics, which can be influenced by both patients and the immediate clinical environment that they are based in. The elements with which pharmacists have agency in changing and interacting with in their clinical environment are described here as proximal environmental factors (3). Distal environmental factors (4) can affect practice policies and pharmacist behaviour, but are outside the agency of clinicians to influence directly. Interventions (5) targeting different factors which affect pharmacists’ navigation of clinical uncertainty when reviewing polypharmacy. Training and education is broadly defined here as formative experiences which can affect how pharmacists rationalise and feel about clinical uncertainty when reviewing polypharmacy. Structured medication reviews were identified as time intensive and directed by policy, but poorly defined as interventions in their own right. The Stopping By Indication Tool and MyComrade were well defined interventions, targeting specific aspects of clinical uncertainty mitigation

The factors identified via thematic analysis, which could affect pharmacists’ perception and management of clinical uncertainty when reviewing polypharmacy (and associated mitigating interventions), are displayed in Fig. 2 and described in the textual narrative below.

### Patient experiences

Relationships with patients were viewed as important to pharmacists, with continuity of care over extended periods (making small iterative changes to prescribed therapy) supporting shared decision-making and deprescribing activity [32, 37, 38]. This patient-pharmacist relationship, previous experiences (including adverse events), understanding of their prescribed medication and disease states, and preconceived expectations of the review were identified as factors that could influence patients’ willingness to engage with a medicine review [38]. 

### Pharmacist experiences

Pharmacists across the included studies expressed various cognitive, emotional and behavioural characteristics in their approach to reviewing polypharmacy and clinical uncertainty. Ramsdale et al.’s focus groups showed pharmacists to be apprehensive over patient judgement or reactions (e.g. anger over suggesting medication changes), and worried about moral judgement from clinician colleagues, such as being judged as ‘stupid’ for asking for advice or disrespectful for suggesting changes to a medication regimen [31]. In spite of these apprehensions, pharmacists generally felt competent to review patients and make alterations to medication regimens within their scope of practice [29, 31]. Pharmacists viewed polypharmacy reviews as complex and time-intensive, with true value only gained if patients were appropriately engaged and involved [30, 35, 37]. 

### Environmental factors

Influences external to the pharmacists and patients directly involved in medication reviews were identified that could either contribute to or mitigate clinical uncertainty. Factors in the immediate environment of practice-based pharmacists, where they had agency to affect them, were classed as ‘proximal,’ with those over which they had no agency or influence classed as ‘distal.’ Proximal environmental factors encompassed healthcare professionals working in the same practice or network. These other clinicians viewed pharmacists as thorough when undertaking reviews and would proactively case find polypharmacy review opportunities if relationships were strong enough [29–31]. Relationships were less strong when pharmacists worked across multiple practice sites, with poor working relations also contributing to clinical inertia (in this article’s context, defined as resistance to making changes to a patient’s prescribed regimen) [29, 32]. Time pressures, clinic structures, organisational structures and managing competing workload were also highlighted as affecting polypharmacy reviews [30, 31, 35]. 

A small number of significant distal environmental factors were also identified. A lack of clear deprescribing evidence or clinical guidelines were frequently cited [30, 37]. Some pharmacists expressed views that undergraduate pharmacy teaching guided pharmacists to interpret guidelines as direct ‘clinical rules.’ [36] It was also noted that a lack of national incentives to review polypharmacy affected the volume of clinical activity dedicated to this [32]. 

### Interventions

Training and education had a significant role to play for the pharmacists involved in focus groups and interviews identified by this review. Learning experiences shaped attitudes towards deprescribing, and pharmacists proactively sought peer support and guidance to mitigate clinical uncertainty [29, 32]. Madden et al.’s interviews showed pharmacists viewing newly appointed pharmacists as seeking clinical knowledge, as opposed to experienced colleagues more familiar with clinical uncertainty and complexity seeking consultation skills development. [36] Some pharmacists also felt that undergraduate pharmacist training was insufficient to deal with the ambiguities that underpin shared clinical decision-making in practice [36]. 

A small number of interventions that potentially mitigated clinical uncertainty for pharmacists were identified in this review. The MyComrade study used GP and pharmacist ‘dyads’ to collaboratively review patients with polypharmacy [35]. These joint, collaborative reviews resulted in an overt decrease in the pharmacist perception of clinical uncertainty, as well as critically appraised and corroborated treatment plans, resulting in enhanced patient (and practitioner) safety [35]. The value of integration within a multidisciplinary team was highlighted in other studies in this review, with pharmacists expressing a desire for more peer support in practice [29, 32]. The Stopping By Indication Tool (SBIT) was developed as an intervention to support clinical pharmacology reviews [33]. This has been identified as a tool which could support deprescribing decision-making in the absence of clear clinical guidelines [33]. 

### Deprescribing and clinical inertia

**Fig. 3:**
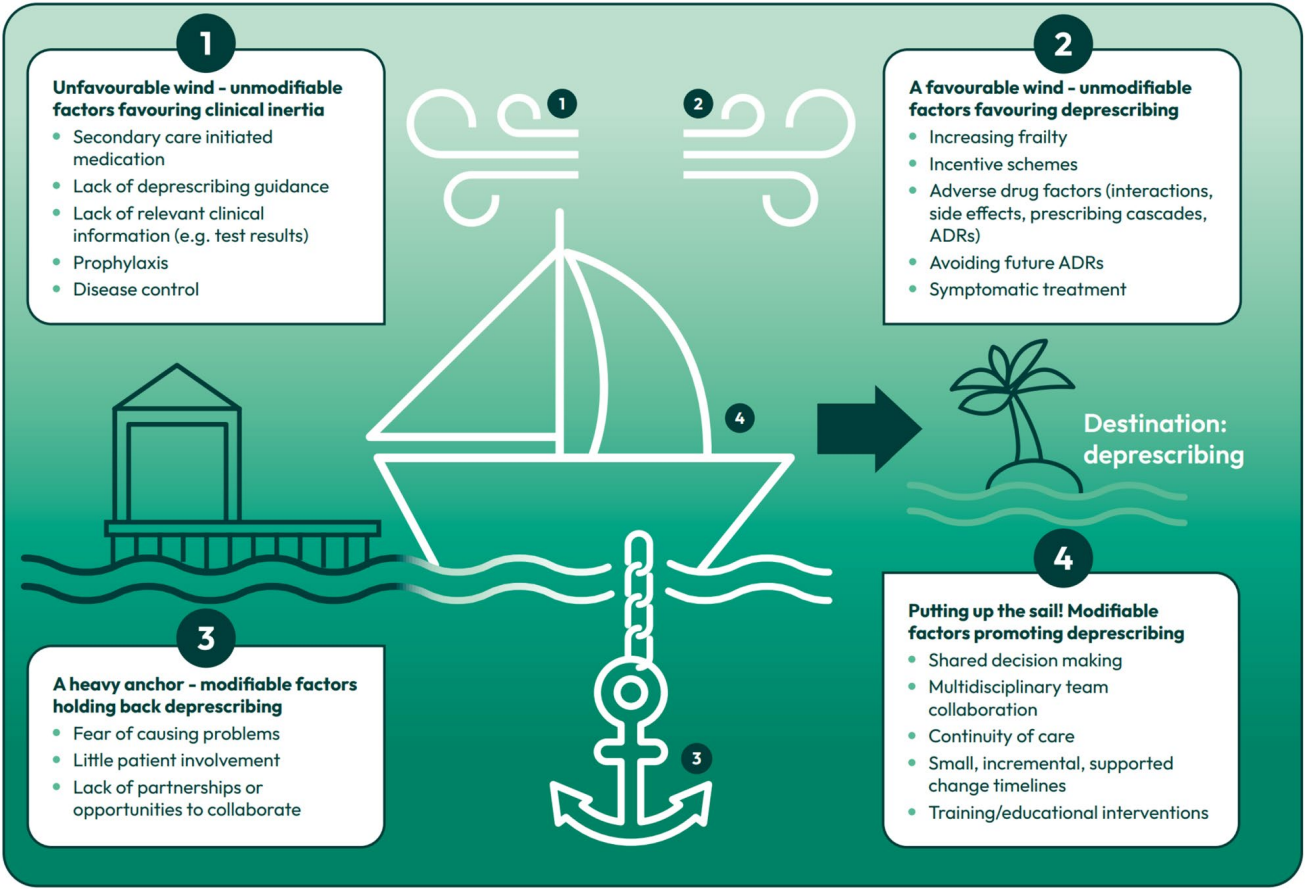
Getting ‘off the dock’ on the journey to deprescribing. This model describes the factors which may encourage or dissuade pharmacists and patients to make a shared decision to deprescribe a medicine. Unmodifiable factors outside of the pharmacist’s agency can either (1) promote clinical inertia (not making any changes to a medicines regimen) and keep the boat on the dock or (2) create a favourable environment for deprescribing and encourage the boat on its journey to the destination of deprescribing. Equally, there are modifiable factors that the pharmacist can interact with which may either (3) hold them back from making a deprescribing decision and keep them anchored to the current medication regimen or (4) promote deprescribing by putting up the sail and embarking on a deprescribing journey with their patient

The decision to deprescribe a medicine was the phenomenon most widely remarked upon by both patients and pharmacists in the included articles. Multiple influencing factors were identified that affected the decision of a pharmacist to either deprescribe or actively take no action and ‘maintain the status quo,’ known as ‘clinical inertia’ [32]. These factors, as described by the included papers, are listed in Fig. 3. The act of deprescribing was characterised as complex and nuanced, with varying considerations to be taken account of for each individual patient. Safety concerns linked to prescribed medication, such as adverse drug effects, drug/disease interactions, side effects and prescribing cascades favoured pharmacists taking proactive measures to make a deprescribing decision [32, 39]. The prescribing rationale also affected clinician perceptions, with pharmacists more likely to take no action for medication prescribed prophylactically or for disease control, in contrast to actively seeking to deprescribe medicines used solely for symptomatic relief [33]. A strong theme across three included papers was a reluctance to change or deprescribe medication initiated in secondary care or by a specialist without prior discussion (described by Charani et al. as ‘prescribing etiquette’). [29, 30, 32, 42] This was further compounded if there was a lack of external relationships or partnerships with the initiating secondary care centre/specialist [32].

Patient characteristics also contributed to pharmacists’ approach to deprescribing. Pharmacists were more comfortable deprescribing for patients with increasing frailty, even in the absence of specific clinical guidance [29, 30]. Proactively involving patients in the deprescribing process and shared decision-making engendered a more favourable environment for deprescribing, whereas clinical activities with no patient involvement were subject to clinical inertia [30, 32]. 

## Discussion

### Summary

A broad range of psychological and socio-cultural factors were identified in this review, which could influence pharmacist decision-making when reviewing polypharmacy in the context of clinical uncertainty. The included articles mainly focussed on the act of deprescribing, which could be reflective of historic roles of practice-based pharmacists, focusing on individual drugs to deprescribe or delivering quality improvement projects. The new expanded role of the primary care clinical pharmacist, independently being able to review, manage and escalate/de-escalate pharmacotherapy autonomously was poorly represented in the currently published literature. The paucity of targeted interventions for clinical pharmacists likely also reflects the novelty of this emerging role in primary care. The conceptual model proposed in this paper is a function of the available literature and therefore provides greater insight into clinical uncertainty into deprescribing, as opposed to the navigation of clinical uncertainty when managing polypharmacy more generally.

### Comparison with existing literature

Organisational structures featured heavily as influencing factors, which reflects the socio-cultural moderators of clinical uncertainty posited by Hillen et al. [14] This has implications for primary care providers in how they structure pharmacist clinics and workload to ensure that appropriate time is given to conducting polypharmacy medication reviews [8, 35]. ‘Tipping the balance’ to make complex prescribing decisions amidst a range of influences has been described in relation to antimicrobial prescribing, which reflects the complexity of factors relating to polypharmacy uncovered by this review [43]. Good inter-disciplinary working and both undergraduate and workplace-based training can improve patient outcomes and enhance the quality of care provided [44, 45]. It is therefore unsurprising that the most overt insight into supporting clinical pharmacists working through clinical uncertainty uncovered in this review was that multidisciplinary working between GPs and pharmacists reduces uncertainty and supports deprescribing in polypharmacy [35]. 

### Implications for practice

Aligning incentives, guidelines and educational interventions towards supporting pharmacists navigate clinical uncertainty when reviewing polypharmacy is important to deliver effective clinical interventions. In England, financial incentives to deliver polypharmacy SMRs in general practice have been de-prioritised in recent years, making the time-intensive work of tackling polypharmacy relatively unattractive to organisations employing clinical pharmacists [46]. Little is recorded in the literature about the value or impact of the primary care postgraduate clinical training schemes that are nationally mandated for primary care pharmacists employed in PCNs. Clinical uncertainty is inherent in the medicines optimisation process for multimorbid patients with polypharmacy. As the trajectory of pharmacy practice moves towards a more clinically focussed profession, developing novel in-practice educational interventions that adequately prepare pharmacists to navigate clinical uncertainty is critical. The introduction of clinical guidelines targeting deprescribing would also support this endeavour [7]. 

### Strengths and limitations

This review was undertaken using a systematic approach, aligning with the definition of a critical literature review [22]. The transparent methodology and routine debriefing of the lead researcher (TK) with the research team (RP, KM, JS) as the review progressed provided opportunities for informal discussion of findings and a forum to explore reflexive insights. Grey literature sources and informally published case studies of practice innovation regarding clinical uncertainty were not interrogated as part of this literature review. These may have contained examples of innovation not captured in academic literature, which may reflect the lag time for formally published articles to be made available regarding this relatively new clinical group of pharmacists. Five of the eleven articles selected for inclusion were identified from citation searching. This review takes a UK perspective of polypharmacy review in English general practice, which may limit the transferability of its findings to other international settings.

## Conclusion

There is limited published literature on how clinical pharmacists manage clinical uncertainty when reviewing polypharmacy in primary care. However, there are various drug, clinician, patient and environmental factors which can affect the decision-making process when pharmacists consider deprescribing in polypharmacy. Multidisciplinary working with GPs and other healthcare professionals can help reduce uncertainty and provide high quality patient care planning. Further research is needed into how pharmacists make decisions when reviewing polypharmacy and what interventions might support their navigation of clinical uncertainty.

## Supplementary Information

Supplementary Material 1

## Data Availability

The datasets (CINAHL, Embase, MEDLINE, PsychInfo)analysed as part of this critical literature review are available through EBSCO (https://www.ebsco.com/and Ovid https://ovidsp.ovid.com/. The search terms used are available in the supplementary information.
