# Supplementary material for: How do pharmacists navigate clinical uncertainty when reviewing polypharmacy? A critical literature review
Source: BMC Prim Care. 2025 Nov 27;26:412. doi: 10.1186/s12875-025-03122-3 (PMC12750690; doi:10.1186/s12875-025-03122-3)
Supplement: Supplementary file 1 — Supplementary Material 1 [file 12875_2025_3122_MOESM1_ESM.docx]

**Supplementary Information**

**Lincoln and Guba’s Evaluative Criteria of Trustworthiness for Qualitative Research**

| **Criterion** | **Detail and Strategies** |
| --- | --- |
| Confirmability | Examines the data, interpretations and findings of the research, as to whether they are internally coherent and supported by the data obtained. Strategies include peer debriefing, reflexive journalling and maintenance of audit trails. |
| Credibility | To ensure that results yielded have sufficient probability that they are credible and that participants who are ‘constructing’ the social reality(s) being interrogated would find the results accurate. Strategies include prolonged engagement in researching phenomenon of interest (to provide scope), persistent observation (to provide depth), participant member checking, deviant case analysis and the use of different methods, investigators, sources and theories triangulated around the research question. |
| Dependability | Examines the process of research and considers replicability and reliability of the research conducted. Strategies include transparently documenting methodological approach, making procedures and/or protocols public, and maintenance of audit trails. |
| Transferability | Examines the ability of the findings of the qualitative research to be ‘transferred’ to other settings. This considers both the original context and the destination context to which the findings may be applied. Strategies include providing contextual information or ‘thick descriptions,’ defining a sampling frame/strategy and providing a sufficient supporting information to enable transferability decisions by the reader of the research. |

Summarised from Lincoln, Y. & Guba, E. Naturalistic Inquiry. (SAGE Publications, Newbury Park, 1985).

**Search Strategy**

Keyword search terms used in MEDLINE, Embase and PsycInfo via OVID:

1 Pharmacists/

2 pharmacist*.ti,ab.

3 pharmacy prof*.ti,ab.

4 chemist.ti,ab.

5 1 or 2 or 3 or 4

6 exp General Practice/

7 Primary Health Care/

8 primary care.ti,ab.

9 pcn.ti,ab.

10 GP.ti,ab.

11 family medic*.ti,ab.

12 ((general or family) adj1 (practice* or practitioner*)).ti,ab.

13 6 or 7 or 8 or 9 or 10 or 11 or 12

14 exp Polypharmacy/

15 polypharm*.ti,ab.

16 ((excess* or multip* or over) adj1 (medicine? or medicat* or prescrib* or prescription* or drug*)).ti,ab.

17 (over adj1 (prescrib* or prescript*)).ti,ab.

18 (over-prescrib* or overprescrib*).ti,ab.

19 (over-prescript* or overprescript*).ti,ab.

20 (poly-pharm* or (poly adj1 pharm*)).ti,ab.

21 14 or 15 or 16 or 17 or 18 or 19 or 20

22 uncertaint*.ti,ab.

23 ((clinic* or medic*) adj1 uncertain*).ti,ab.

24 ((clinic* or medic*) adj1 ambig*).ti,ab.

25 unpredictab*.ti,ab.

26 exp Uncertainty/

27 unclear*.ti,ab.

28 22 or 23 or 24 or 25 or 26 or 27

29 exp "Medication Review"/

30 ((med* or therap* or drug*) adj2 (review* or counsel* or management)).ti,ab.

31 (deprescr* or de-prescr*).ti,ab.

32 exp Deprescriptions/

33 29 or 30 or 31 or 32

34 exp Decision Making/

35 (decision-making or "decision making").ti,ab.

36 (clinical* adj1 decision).ti,ab.

37 ((clinical or practice) adj2 behaviour).ti,ab.

38 34 or 35 or 36 or 37

39 5 and 13 and 21 and 33 and 38

40 5 and 13 and 21 and (33 or 38)

41 5 and 13 and 28 and 33 and 38

42 5 and 13 and 28 and (33 or 38)

43 5 and 13 and 21 and 28 and (33 or 38)

44 5 and 13 and 21 and 28 and 33 and 38

These search terms were mirrored line by line for searching the CINAHL database via EBSCO. Searches 39 – 44 were the final searches used to inform the literature review.
